# Death of Dr. Satterwhite

**Published:** 1841-11

**Authors:** 


### DEATH OF DR. SATTERWHITE.

The name and worth of this physician are familiar to all who ever passed much time at Lexington in this State, for he was for twenty years engaged in that city in an extensive and reputable practice. We remember him as he was eighteen years ago, a young physician who had gained the confidence of that intelligent community, as a judicious, kind, attentive practitioner and conscientious man. This character Dr. Satterwhite sustained to the time of his death, which occurred on the 23d of October, suddenly, in consequence of a fall from his horse. The animal became frightened, reared and fell backwards, and in the fall dislocated the neck of the rider. Dr. Satterwhite was a member of the Methodist Episcopal Church, and his life was one of exemplary piety. His example is instructive to the young physician. It proves how much may be done by industry and a virtuous life towards securing the highest popularity in our profession, with its attendant advantages of wealth and independence, unaided by any extraordinary mental endowments. Y.

